# Clinicopathological and imaging features of pulmonary alveolar microlithiasis in a dog – a case report

**DOI:** 10.1186/s12917-020-02593-z

**Published:** 2020-10-07

**Authors:** Ana Canadas Sousa, Joana C. Santos, Clara Landolt, Catarina Gomes, Patrícia Dias-Pereira, Cláudia S. Baptista

**Affiliations:** 1 Department of Molecular Pathology and Immunology, Veterinary Pathology Laboratory, Institute of Biomedical Sciences Abel Salazar - University of Porto (ICBAS-UP), Rua Jorge Viterbo Ferreira 228, 4050-31 Porto, Portugal; 2 Department of Veterinary Clinics, Institute of Biomedical Sciences Abel Salazar - University of Porto (ICBAS- UP), Veterinary Hospital of the University of Porto (UPVet), Rua Jorge Viterbo Ferreira 228, 4050-313 Porto, Portugal; 3 Centro de Estudos de Ciência Animal (ICETA-CECA), Instituto de Ciências e Tecnologias Agrárias e Agro-Alimentares da Universidade do Porto, Rua D. Manuel II, Apartado, 55412, 4051-401 Porto, Portugal

**Keywords:** Pulmonary alveolar microlithiasis, Canine, Radiology, CT, Histopathology

## Abstract

**Background:**

The aetiology of pulmonary alveolar microlithiasis (PAM) in animals is still unknown. In humans, this pulmonary disorder is a rare autosomal recessive disorder triggered by a mutation in the gene SLC34A2, which causes deposition and aggregation of calcium and phosphate in the pulmonary parenchyma with formation of microliths. Although histopathological examination is required for a definite diagnosis, in humans, imaging modalities such as computed tomography can demonstrate typical patterns of the disease. This is the first description of the computed tomographic (CT) features of a histologically confirmed PAM in dogs.

**Case presentation:**

The following report describes a case of a 7-year-old female Boxer dog evaluated for paroxysmal loss of muscle tone and consciousness with excitement. The main differential diagnoses considered were syncope, seizures, and narcolepsy-cataplexy. The results of the complete blood count, serum biochemistry panel, urinalysis, arterial blood pressure, echocardiography, abdominal ultrasound, Holter monitoring, and ECG were all within normal limits. Additional exams included thoracic radiographs, head and thorax CT, bronchoalveolar lavage (BAL), and CT-guided cytology. Thoracic radiographs revealed micronodular calcifications in the lungs, with sandstorm appearance. Computed tomography of the thorax showed the presence of numerous mineralized high-density agglomerates of multiple sizes throughout the pulmonary parenchyma, a reticular pattern with ground glass opacity and intense mineralized fibrosis of the pleural lining. Head CT was unremarkable. BAL and CT-guided cytology were inconclusive, but imaging features strongly suggest the diagnosis of PAM, which was histologically confirmed after necropsy.

**Conclusions:**

This case report contributes to the clinicopathological and imaging characterization of pulmonary alveolar microlithiasis in dogs. In this species, the diagnosis of PAM should be considered when CT features evidence a reticular pattern with ground glass opacity and the presence of an elevated number and size of calcifications.

## Background

In humans, pulmonary alveolar microlithiasis (PAM) is a rare autosomal recessive disorder [1] caused by a mutation in the gene SLC34A2 that encodes a sodium-dependent phosphate co-transporter, responsible for the clearance of phospholipids of the alveolar space. The mutation is accountable for biochemical abnormalities of the alveolar type II cells, causing deposition and aggregation of calcium and phosphate in the pulmonary parenchyma with formation of microliths [2].

In veterinary medicine, PAM is also a very rare condition. The first documented case in dogs was reported by Liu and co-workers in 1969 [3]. Further reports about this disease in a sheep [4], dogs [3, 5–9], a cat [10], and exotic and wild animals [11–15] were also described. Nevertheless, the aetiology of this pulmonary disorder in animals is still unknown [16].

As in humans, PAM is usually detected incidentally during routine examinations, since patients with this disease are frequently asymptomatic at the time of diagnosis [8]. However, clinical course ranges from asymptomatic to fatal [17]. Although a histopathological examination is required for a definite diagnosis, thoracic imaging with high-resolution computed tomography (HRCT) can demonstrate typical patterns of the pulmonary disease [1].

To the author’s knowledge, there are no publications describing the computed tomographic features of pulmonary alveolar microlithiasis in dogs. Accordingly, the aim of this case report is to describe the clinicopathological and the computed tomographic features of a histologically confirmed clinical case of PAM in order to contribute to the characterization of this rare disease.

## Case presentation

A 7-year-old neutered female Boxer was presented for evaluation of paroxysmal acute loss of muscle tone and consciousness with rapid recovery to normal mentation, precipitated by strong positive emotions such as the owner’s arrival and playing. The first episode was observed 1 week prior to presentation (at least 5 episodes per day). These events lasted from a few seconds to one minute, during which the limbs were mainly flaccid; sometimes the animal was able to stand and walk normally immediately after. On the day of presentation, physical examination was normal, including the respiratory evaluation and the neurological exam. The main differential diagnoses considered were syncope, seizures, and narcolepsy-cataplexy. The results of the complete blood count, serum biochemistry panel (urea, creatinine, alanine aminotransferase, alkaline phosphatase, glucose, total proteins, albumin, globulins, albumin/globulin ratio and amylase), urinalysis, arterial blood pressure, echocardiography, abdominal ultrasound, 24 h Holter monitoring, and ECG were all within normal limits. Additional exams included thoracic radiographs, head and thorax CT, bronchoalveolar lavage (BAL), and a CT-guided cytology.

**Fig. 1:**
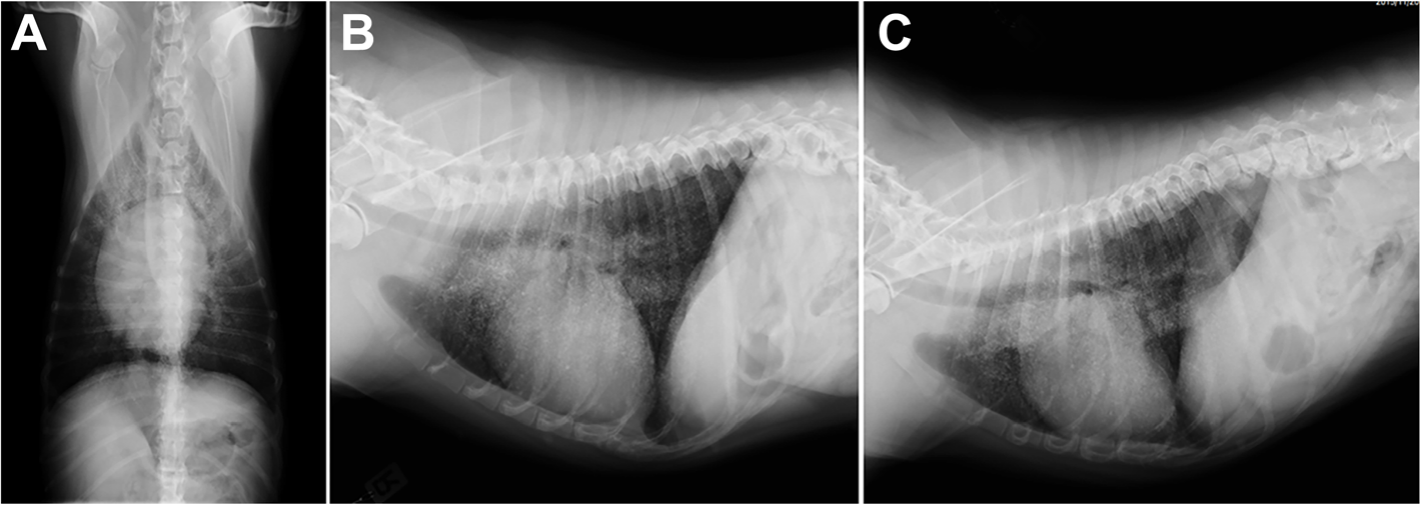
**a** Ventrodorsal, **b** right and **c** left lateral thoracic radiographs. We can observe diffuse micronodular calcified opacities with a greater concentration in cranial lung lobes, with a sandstorm appearance. The outlines of the heart and diaphragm are clearly visible. Generalized spondylosis of the thoracic vertebra

Ventrodorsal, left and right lateral radiographs of the thorax were performed (Philips Optimus 50, Philips Portuguesa S.A., Porto Salvo, Portugal, 86 kVp and 78 kVp respectively, 6 mAs) using a computed radiography system (Fujifilm FCR Prima, Fujifilm Europe GmbH, Vila Nova de Gaia, Portugal) (day of presentation). Thoracic examination evidenced micronodular mineral opacities predominantly localized in both cranial lung lobes, with a sandstorm appearance (Fig. 1). Multifocal bridging thoracic spondylosis deformans was also observed. The remaining intrathoracic structures were within normal limits.

**Fig. 2:**
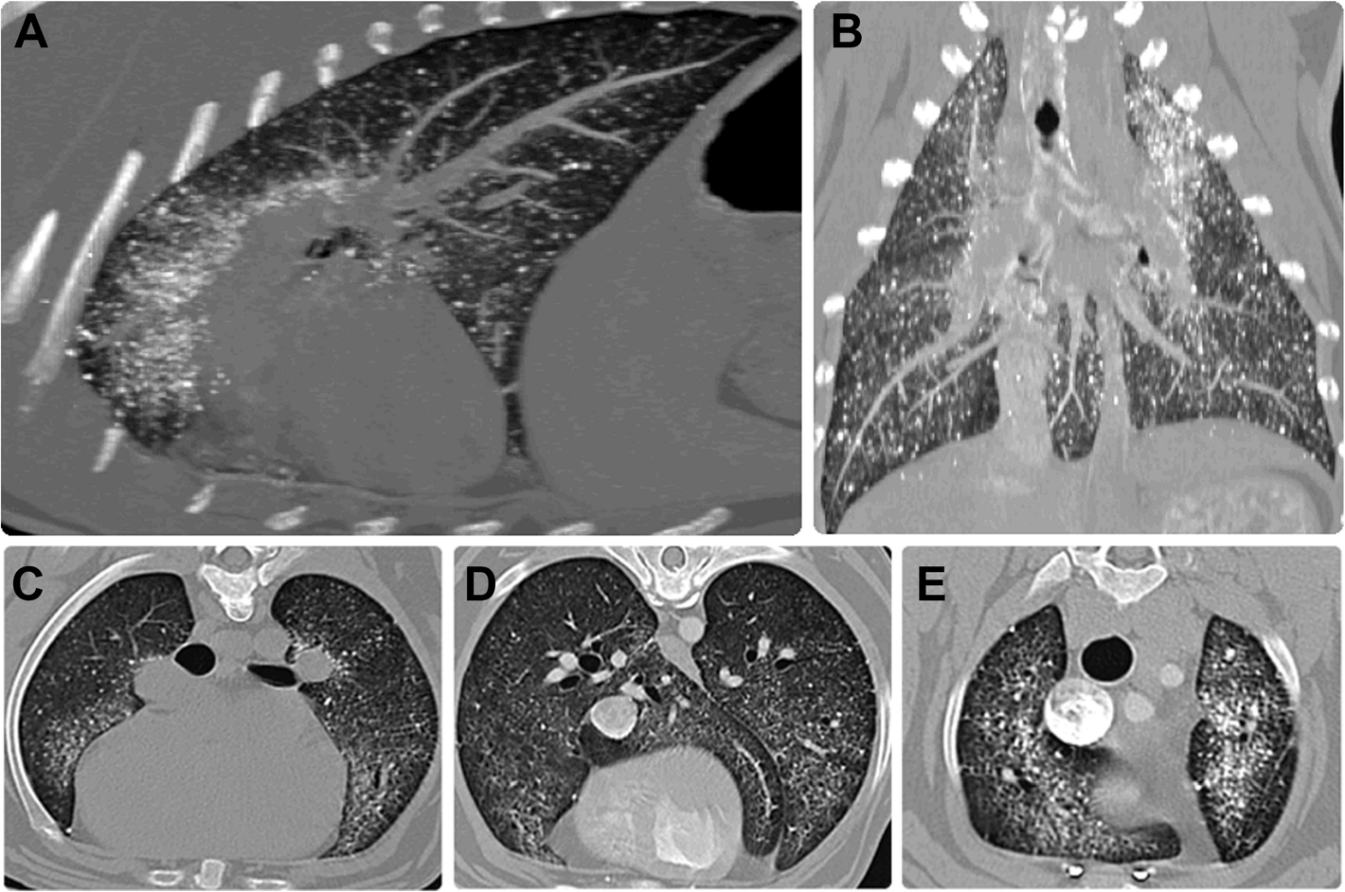
CT volumetric reconstruction MIP (**a** and **b**) highlighting the pulmonary microliths distribution. The animal is positioned in sternal recumbency. Pre (**c**) and postcontrast (**d** and **e**) CT scans evidencing numerous non-contrast enhancing, mineralized high-density agglomerates of multiple sizes (up to 3.8 mm) throughout the parenchyma. A reticular pattern with superimposed ground glass opacity (*) with clear bronchial and vascular margins and thickening of the pleural lining (arrow) is also visible

Computed tomography (8-slices General Electric LightSpeed, General Electric Healthcare, Carnaxide, Portugal) of the head and thorax was acquired before and after intravenous injection of iodinated contrast medium (Iopramide, Ultravist 370, Schering, Berlin, Germany, at 2 ml/kg; 769 mgI/ml) administered manually via cephalic vein catheter (dual phase protocol). The animal was pre-medicated with methadone (0.2 mg/kg, intramuscular administration), anesthetized with propofol (4 mg/kg, intravenous administration) and maintained with isoflurane supplied with oxygen and positioned in sternal recumbency. Images were acquired using the helical scan mode, with 1.3–2.5 mm slice thickness, bone, soft tissue, and lung algorithms. Other CT technical parameters were 120 kV tube voltage, 190 mAs tube current, 0.8 s tube rotation time, and reconstructions interval with 50% overlap for the thorax and head. The displayed field of view was 32.5 cm, and the image matrix was 512 × 512. Multiplanar reconstructions, as well as maximal intensity projection (MIP) (Fig. 2a-b) and minimal intensity projection reconstructions were also performed. Numerous mineral attenuating (average 600 HU), non-contrast enhancing nodules were noted spread throughout the pulmonary parenchyma, more pronounced in both cranial lung lobes (Fig. 2c and e). We could also observe a diffuse reticular pattern and thickening of the pleural lining. A ground glass opacity with clear bronchial and vascular margins was visible in the caudal right, caudal left, and accessory lobes (Fig. 2e). Mineral deposition was not observed in any other organ or tissue scanned. A BAL (direct smears and cytospin) and a CT-guided cytology were performed evidencing a nonspecific inflammation with increased mucus production; the pulmonary parenchyma highlights the increase of macrophages that can translate an immune response to a foreign agent/body/substance/cell (maybe mineralization/calcification). Head CT scans were unremarkable. Accordingly, treatment was initiated to rule out acquired narcolepsy-cataplexy with imipramine (4 mg/kg once daily, oral administration). No treatment plan for the pulmonary disorder was established. After day 20, the clinical condition deteriorated, with increased frequency and severity of syncopal episodes. After day 27, cyanosis, tachypnoea, and exercise intolerance were reported by the owners. While they were absent, the animal died at home 30 days post presentation.

**Fig. 3:**
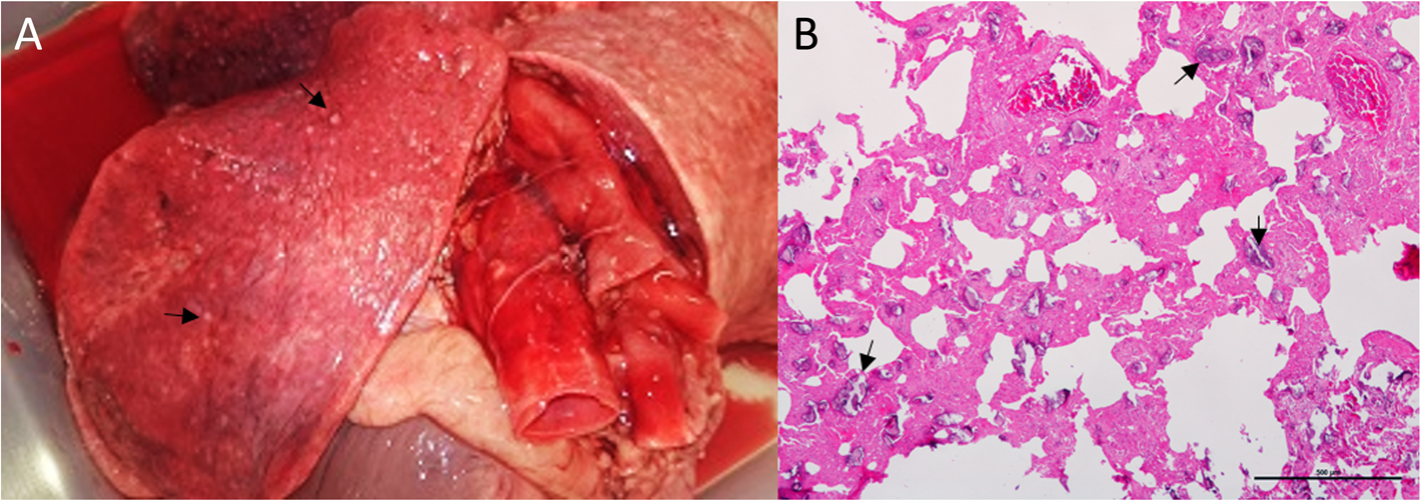
**a** Macroscopic view and **b** microscopic image of the lung. **a** Pinpoint white, round, and slightly elevated foci are visible, along with a pale grey colour on the lung's surface (arrows). **b** Marked thickening of interstitial tissue and interalveolar septa, reduction of alveolar space, and variably sized microliths mostly located at the alveolar walls (arrows). HE. Bar 500µm

After the owner’s consent, post-mortem examination was performed. Chronic respiratory disease was confirmed, revealing lung firmness with reduced elasticity, and showing a pale grey-pink colour and patchy emphysema (Fig. 3a). Numerous diffuse palpable foci of mineralization were detected in the lungs, giving the organ a fine grainy consistency. A gritty texture was evident on the cut section. No major pathological abnormalities were found in any other organ.

Lung samples were collected for histological diagnosis. Samples, routinely processed and paraffin-embedded, were fixed in 10% buffered formalin, sectioned at 3 µm, and stained with haematoxylin and eosin (HE) for routine microscopic examination. Selected areas of the lung were also stained with periodic acid-Schiff (PAS), Prussian blue, and Masson’s trichrome.

A distortion of the pulmonary architecture was observed under microscopic examination, characterized by marked thickening of pleura, interstitial tissue, and alveolar septa, along with a significant reduction of the alveolar space (Fig. 3b). Areas of alveolar emphysema, characterized by distension and destruction of the alveolar walls, were also found. Numerous circular to irregularly shaped, amorphous basophilic concretions (microliths) were detected widespread across the pulmonary parenchyma. These variably sized, PAS positive structures, which exhibited concentric lamination, were located at the alveolar walls or free within the alveolar space (intra-alveolar lamellar microliths) (Fig. 3b). Subpleural and interstitial fibrosis was evidenced by Masson trichrome stain. Prussian Blue stain was negative, excluding the hypothesis of iron deposition. No signs of an inflammatory response were detected.

## Discussion and conclusions

In humans, PAM is characterized by an insidious clinical course, eventually leading to respiratory failure with hypoxemia, “cor pulmonale”, or even cardiac failure. Infection, low temperatures, smoking, and inhalation of environmental substances can also accelerate the progression of the disease [1, 5]. In this case report, imaging features and the presence of microliths, subpleural, and interstitial fibrosis at necropsy support the diagnosis of PAM. Considering the initial clinical presentation, the main differential diagnoses pondered were syncope (cardiovascular, neurally mediated, respiratory, hypoglycaemia), seizures (metabolic, central nervous system - CNS) and narcolepsy-cataplexy. As this dog was a boxer, the main focus was to rule out arrhythmogenic right ventricular cardiomyopathy (ARVC), a common condition in this breed, characterized by serious ventricular arrhythmias, syncope, and high risk of sudden death [18, 19]. Nevertheless, arterial blood pressure, echocardiography, ECG, and 24 h Holter monitoring were all within normal limits.

In humans, the variable individual chest radiographs and HRCTs features of the disease have been amply described, enabling a radiological classification subdivided into four evolutionary phases, according to different degrees of severity [20, 21]:

1. First phase, small number and precalcified microliths; PAM is not recognized by radiological features; patients most likely asymptomatic;
2. Second phase, the lungs appear “sandy”, featuring diffuse, scattered calcified micronodules with a diameter of < 1 mm; typical radiological picture; the outlines of the heart and diaphragm are still clearly visible; in some cases, the single micronodules show a larger diameter, ∼2–4 mm, and resemble berries;
3. Third phase, the number and volume of the opacifications increases; the image becomes more granular, nodular, and confusing, due to the initial thickening of the interstitial weave, which partly masks the micronodules; in the medial and inferior fields, a superimposition of the opacifications hides the outlines of the heart and diaphragm;
4. Fourth phase, the largest number and size of the calcified deposits; interstitial calcified fibrosis occurs; there may be paraseptal emphysema, large bubbles, or air cysts in the upper lobes, as well as pneumothorax and areas of ossification.

In dogs with PAM, a pulmonary miliary pattern due to mineralization has been described in thoracic radiographs of animals with respiratory clinical signs [3, 7, 22]. However, other diseases associated with this pulmonary pattern such as hemosiderosis, pneumoconiosis, amyloidosis, fungal pneumonia, metastatic and dystrophic calcification (chronic renal failure, hyperadrenocorticism, hyperparathyroidism, vitamin D poisoning) should also be pondered as differential diagnoses [23]. In this dog, the complete blood count, serum biochemistry panel (including renal function), urinalysis, and abdominal ultrasound were within normal limits (no evidence of dystrophic calcifications, adrenomegaly, or systemic signs of fungal disease – also supported by the BAL and necropsy negative results for fungal hyphae). Additionally, the animal had no history of vitamin D ingestion or exposure to both foreign antigens and inorganic triggers of inflammation that promote an exuberant granulomatous immune response, so the previous differential diagnoses were considered unlikely.

In humans, a “crazy paving” pattern on the chest HRCT with calcifications along the interlobular septa may be considered diagnostic, even pathognomonic, of the third and fourth phases of PAM [21]. High resolution CT scans were not obtained for this dog. Nevertheless, computed tomography evidenced a reticular pattern with micronodulation of mineralized densities (highlighted by MIP volumetric reconstruction) (Fig. 2a-b). The demonstration of microliths in the bronchoalveolar lavage, under CT scan guidance, is an effective way to diagnose PAM [17]. However, a BAL was performed on this dog, but the result was inconclusive to diagnose this condition.

To exclude central nervous system abnormalities, such as tumours, inflammation, and infections (viral, bacterial, fungal, parasitic), a CT scan of the head was also obtained. Although magnetic resonance imaging is preferred whenever disease of the CNS is suspected, CT was readily accessible since the animal also had clinical indication for a CT of the thorax (in order to scrutinize lesions identified in thoracic radiographs). Since the head CT was within normal limits, treatment with imipramine was initiated to rule out acquired narcolepsy-cataplexy.

In humans, this disease is usually detected incidentally during routine examinations, since patients with PAM are frequently asymptomatic at the time of diagnosis, evidencing a clinical-radiological dissociation [23]. In this dog, when imaging procedures were performed, there was no evidence of respiratory clinical signs. Accordingly, we theorize that narcolepsy-catalepsy precipitated the initial syncopal episodes which led to the detection of PAM imaging findings. Nevertheless, the cause of sudden death is unknown. While ARVC was not reported on histopathology, it cannot be ruled out in this dog. The cause of respiratory distress might have been due to hypoxaemia caused by pulmonary fibrosis, pulmonary thromboembolism, or unreported chronic changes within the interstitial lungs [24]. Hypoxia can be an underlying metabolic cause of ventricular arrhythmia leading to sudden death [25]. It is important to note that imaging features were observed on day 1 (thoracic radiographs) and day 13 (head and thorax CT), and the necropsy was performed on day 30. The previous timeline may explain the differences in the evolutionary phase between imaging modalities and findings at necropsy.

At the microscopic level, PAM should be differentiated from broncholithiasis, in which mineralized concretions fill the airways, not alveoli [26], and from metastatic and dystrophic calcification, where mineral deposits are found in the pulmonary interstitium or in areas of necrosis, respectively [5, 27]. Contrary to other documented veterinary cases [10, 13], inflammation was not associated with the presence of microliths in this dog, similarly to what has been shown in humans. As described, to varying degrees and in all reported canine and feline cases of PAM [3, 5–8, 10], we also observed pulmonary interstitial and subpleural fibrosis.

There is still no effective medical or gene therapy for PAM. Systemic corticosteroids, calcium-chelating agents, diphosphonates, and serial bronchopulmonary lavage have been shown to be ineffective and are used as palliative treatments [17]. Lung transplantation, at some stage of the disease, is the elective treatment in human medicine [28]. In veterinary patients, this surgical procedure was only performed in animal models, including cats and dogs, for investigation purposes [29–31]. The dog here reported was not submitted to any palliative treatment for PAM.

As in humans, individual cases of PAM may allow to establish a radiological classification according to the severity of the disease. Based on our review of the literature, this is the first report describing the computed tomographic features of pulmonary alveolar microlithiasis in dogs. The hallmark of this disease is a clinical-radiological dissociation, so it is possible that PAM is being underdiagnosed in veterinary medicine. In dogs, the diagnosis of PAM should be considered when CT scans evidence a reticular pattern with ground glass opacity and the presence of an elevated number and size of calcifications.

## Data Availability

All abnormal data obtained during this study is included in this published article. The data found to be within normal limits is available upon reasonable request to the corresponding author.

## Abbreviations

BAL: Bronchoalveolar lavage
CNS: Central nervous system
CT: Computed tomography
ECG: Electrocardiography
HE: Haematoxylin and eosin stain
HRCT: High-resolution computed tomography
HU: Hounsfield unit
MIP: Maximal intensity projection
PAM: Pulmonary alveolar microlithiasis
PAS: Periodic acid-Schiff
